# Gelatinization and Pasting Property of Small Granular Starch from *Chlamydomonas reinhardtii* and Its Structural Basis

**DOI:** 10.3390/gels12030241

**Published:** 2026-03-13

**Authors:** Tao Xu, Yongheng Zhong, Wei Jiang, Xuan Luo, Xiaofang Zhou, Peiwu Li

**Affiliations:** 1 Xianghu Laboratory, Hangzhou 311231, China; 2 College of Biosystems Engineering and Food Science, Zhejiang University, Hangzhou 310058, China; 3 College of Life Sciences, Shanghai Normal University, Shanghai 201400, China

**Keywords:** *Chlamydomonas reinhardtii* starch, gelatinization property, pasting property, multiscale structure, terrestrial plant starch, digestibility

## Abstract

The gelatinization and pasting behavior of starch play a critical role in governing its suitability for various food and non-food applications. Although *Chlamydomonas reinhardtii* is the most-studied microalga, its starch gelatinization and pasting properties have remained elusive. In this study, we applied nitrogen limitation to promote the starch accumulation of *C. reinhardtii* and recovered the starch using high-pressure homogenization. The multiscale structure and properties of *C. reinhardtii* starch (CRS) were comprehensively analyzed and compared with those of commonly used terrestrial plant starch. Results showed that CRS possesses a unique multiscale structure characterized by an exceptionally high degree of branching (18.6%) and a thinner crystalline lamellae (9.29 nm). While maintaining an A-type crystalline pattern, CRS granules exhibited higher crystallinity compared with other microalgal starches. CRS had an irregular red blood cell-like morphology with a small size (~1 μm diameter). Physicochemical analysis revealed that CRS has an intermediate gelatinization temperature and a pasting profile defined by low viscosity and remarkable shear resistance, suggesting high stability during hydrothermal processing. Significantly, cooked CRS demonstrated a lower hydrolysis rate and higher resistant starch content than several common terrestrial starches. It is attributed to its higher degree of branching and superior thermostability. This study extends the fundamental knowledge of CRS and provides a critical scientific basis for its application as a novel, sustainable ingredient with special gel properties in the future food industry.

## 1. Introduction

The global food system is currently facing unprecedented pressure due to rapid population growth, climate change, and the shrinking availability of arable land [1]. These challenges have catalyzed a shift toward sustainable, alternative bio-resources. Among these, microalgae have garnered significant attention for their exceptionally high photosynthetic efficiency, converting solar energy and carbon dioxide into biomass at rates ten to fifty times higher than traditional terrestrial crops [2]. Furthermore, their cultivation does not compete with food crops for fertile soil, as they can be grown in photobioreactors. While initial research primarily focused on microalgae for biofuels [3], the focus has pivoted toward their application in the food industry [4], driven by their rich profiles of proteins [5], polyunsaturated fatty acids [6,7], and carbohydrates [8].

Within the diverse kingdom of microalgae, *Chlamydomonas reinhardtii* stands as the most extensively characterized model organism [9,10]. Its genome has been fully sequenced, and its metabolic pathways are well-mapped, making it a “green yeast” for biotechnological advancement [11]. Despite its prominence in laboratory research, the industrial utilization of its primary storage polysaccharide, starch, remains in its infancy [8]. In *C. reinhardtii*, starch is synthesized within the chloroplast and stored as insoluble granules, serving as a critical energy reserve during periods of environmental flux [12,13]. It is synthesized during the daytime and consumed at night [14]. Thus, in natural conditions, starch content is relatively low compared with protein [15]. While the proteomic and lipidomic profiles of this species have been explored in depth [16,17], the structural and functional differences in its starch fraction have been largely overlooked.

The accumulation of starch in microalgae is a highly plastic process, sensitive to environmental stimuli [18,19]. Among various cultivation strategies, nitrogen limitation is recognized as one of the most effective triggers for starch hyper-accumulation [20]. Under nitrogen-replete conditions, microalgae prioritize protein synthesis and cellular division. However, when nitrogen becomes scarce, the cellular carbon flux is redirected from protein synthesis toward the accumulation of energy-dense carbon compounds [14,21]. In *C. reinhardtii*, this metabolic shift leads to a dramatic increase in starch granule density within the pyrenoid and stroma of the chloroplast [22]. Understanding how this stress-induced synthesis affects the internal architecture of the starch granule is essential for predicting its behavior in food processing.

To fully utilize *C. reinhardtii* starch (CRS) in food systems, its multiscale structure must be elucidated. Starch is not a simple polymer but a complex hierarchy: from the molecular level (amylose and amylopectin chains) and the crystalline/amorphous lamellae (9–10 nm) to the growth rings and the whole granule morphology (micrometer scale) [23]. These structural features collectively determine the starch’s physicochemical properties, such as gelatinization temperature, viscosity, and digestibility [24]. Most research focused on starch synthesis gene function and starch production promotion in *C. reinhardtii*. The multiscale structure of CRS is rarely reported, among which the molecular structure is the most frequently reported. Under nitrogen supply conditions, CRS has less than 5% amylose. While nitrogen deprivation greatly increased amylose content (ranging from 15 to 35%) [25]. The chain length distribution of debranched CRS showed a peak at 10–11 [19,25,26]. The X-Ray diffraction (XRD) spectrum of CRS showed A-type crystal structure patterns and crystallinity of 28% [27] and 40% [28] were reported. The starch granule of *C. reinhardtii* is small compared with most crop starches, with a diameter of about 1 μm [29]. Most of these structural analyses were done in *C. reinhardtii* strain 137C, and multiscale structures such as short-range ordered structure, lamellar structure, and degree of branching have not been characterized [13,18,19,20,26,27,29,30,31].

Starch properties such as gelatinization, pasting, and digestibility are major determinants for its application [32]. Gelatinization and pasting properties are important phenomena that affect the cooking properties, texture, and palatability of starch-based food products [33,34]. The digestive properties of starch are of paramount importance in human nutrition. The rate and extent of starch hydrolysis in the small intestine are influenced by the granule’s surface area, porosity, and crystalline polymorph (A, B, or C-type) [24]. While terrestrial plant starches, such as corn and potato, have been categorized extensively [35], the properties of CRS remain ambiguous.

This study aims to fill the knowledge gap regarding the structural-functional relationships of CRS. By employing nitrogen limitation to promote starch accumulation of *C. reinhardtii* and high-pressure homogenization for starch recovery, we provide a comprehensive analysis of the multiscale structure and digestive profiles of CRS. Furthermore, this research provided a direct comparison of CRS with commonly used terrestrial plant starches. The findings of this study not only extend our fundamental understanding of microalgal polysaccharides but also provide a critical scientific basis for the application of CRS as a novel, sustainable ingredient in the future food industry.

## 2. Results and Discussion

### 2.1. Molecular Structure

**Figure 1:**
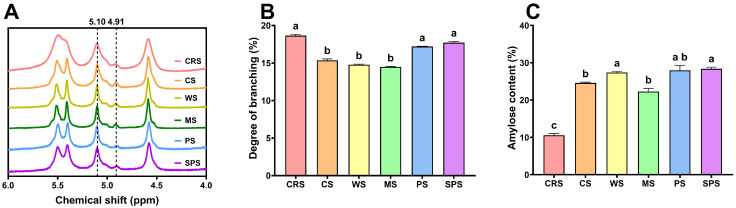
The ^1^H NMR spectra (**A**), degree of branching (**B**), and amylose content (**C**) of *Chlamydomonas reinhardtii* starch (CRS), cassava starch (CS), wheat starch (WS), maize starch (MS), potato starch (PS), and sweet potato starch (SPS). Bars that do not share the same lowercase letter are significantly different (*p* < 0.05).

The degree of branching of starch samples was shown in Figure 1. Figure 1A shows the proton nuclear magnetic resonance (^1^H NMR) spectra of starch samples. Clear peaks at 5.10 ppm and 4.91 ppm were seen for all starches, which represented the α-1,4 and α-1,6 glycosidic bonds, respectively. The area of these two peaks was applied to calculate the degree of branching of starch samples. CRS presented the highest degree of branching (18.6%), significantly higher than CS, WS, and MS, but statistically the same as PS and SPS. The higher degree of branching may come from the lower content of amylose or more branching points inside amylopectin. A high degree of branching indicates CRS may have a low peak viscosity in pasting [36]. Additionally, the crystallinity of CRS is likely to be relatively high [37]. The amylose content of CRS was significantly lower than that of other starches (Figure 1C), consistent with the higher degree of branching. The low amylose content of CRS indicated it may have a low gelatinization enthalpy [38].

### 2.2. Short-Range Ordered Structure

**Figure 2:**
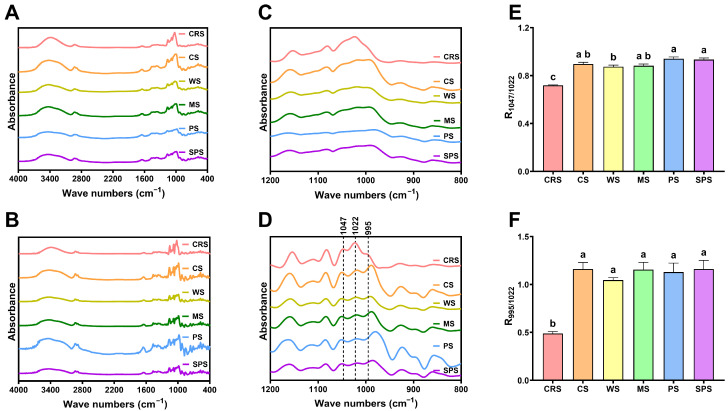
The FTIR (**A**,**B**) and deconvoluted (**C**,**D**) spectra and absorbance ratio (**E**,**F**) of *Chlamydomonas reinhardtii* starch (CRS), cassava starch (CS), wheat starch (WS), maize starch (MS), potato starch (PS), and sweet potato starch (SPS). Bars that do not share the same lowercase letter are significantly different (*p* < 0.05).

The short-range ordered structure was analyzed by Fourier-transform infrared spectroscopy (FTIR). The spectra of starch samples are shown in Figure 2A. The spectrum of CRS had similar peaks at 3500–3000 cm^−1^, 3000–2800 cm^−1^, and 1700–1000 cm^−1^ to other starch samples, which represent the stretching of O-H, C-H, C-O, C-C, and C-O-H bonds in starch structure [39]. Figure 2B presents the deconvoluted spectra, and spectra between 1200 and 800 cm^−1^ can be seen clearly in Figure 2D. The deconvoluted spectra exhibited three peaks at about 1047, 1022, and 995 cm^−1^, while no clear peaks were found in the original spectra (Figure 2C). The absorbance at 1047 and 995 cm^−1^ is recognized as an ordered structure, and 1022 cm^−1^ represents an amorphous structure [40,41]. The ratio of absorbance at these wave numbers, R_1047/1022_ and R_995/1022_, was compared between starch samples (Figure 2E,F). CRS showed significantly lower ratios than terrestrial plant starches. Jiang et al. [39] found similar results when analyzing starch from another microalgae, *Chlorella sorokiniana*. Figure 2D showed that elevated absorbance at 1022 cm^−1^ and decreased absorbance at 995 cm^−1^ were accounted for by the lower R_1047/1022_ and R_995/1022_ of CRS, respectively. This indicated that microalgal starch has a higher amorphous region and a lower crystalline region compared with terrestrial plant starch.

### 2.3. Lamellar Structure

**Figure 3:**
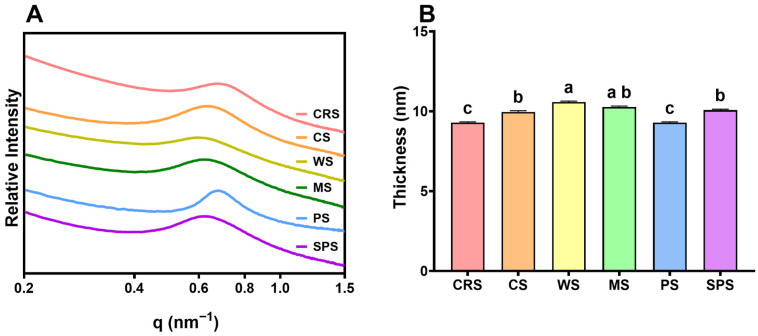
The SAXS curve (**A**) and lamellar thickness (**B**) of *Chlamydomonas reinhardtii* starch (CRS), cassava starch (CS), wheat starch (WS), maize starch (MS), potato starch (PS), and sweet potato starch (SPS). Bars that do not share the same lowercase letter are significantly different (*p* < 0.05).

To further determine the key region lowering the CRS short-range ordered structure, the lamellar thickness was determined by small-angle X-ray scattering (SAXS) [42]. The SAXS curve showed different peaks for starch samples between 0.6 and 0.8 nm^−1^ (Figure 3). All samples had a broad peak from 0.4 to 1.0 nm^−1^ except PS. The average lamellar thickness of CRS was 9.29 nm, highly consistent with *Chlorella sorokiniana* starch (9.28 nm) reported by Jiang et al. [39]. CRS lamellar thickness was significantly lower than CS, WS, MS, and SPS, consistent with Jiang et al. [39]. Combining the lower short-range ordered structure determined by FTIR (Figure 2E,F), the lower lamellar thickness indicated a thinner crystalline layer of CRS compared with other starches.

### 2.4. Crystalline Structure

**Figure 4:**
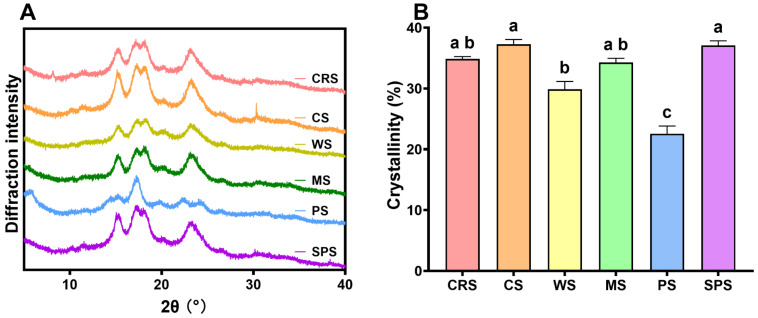
The XRD pattern (**A**) and crystallinity (**B**) of *Chlamydomonas reinhardtii* starch (CRS), cassava starch (CS), wheat starch (WS), maize starch (MS), potato starch (PS), and sweet potato starch (SPS). Bars that do not share the same lowercase letter are significantly different (*p* < 0.05).

XRD was applied to directly determine the crystalline type and crystallinity of CRS. Figure 4A presents the XRD patterns of starch samples. CRS exhibited the same typical A-type crystalline diffraction peaks at about 15.2°, 17.2°, 18.1°, and 23.1° (2θ) [43] as CS, WS, MS, and SPS. Only PS showed B-type crystalline pattern with peaks at 5.6°, 17.2°, 22.4°, and 24.2° [44]. Previous studies also found that *C. reinhardtii* 137C produced starch with A-type crystalline structure [26,27,28]. Starch from other microalgae was found to be all A-type crystalline structure so far [39,45,46,47]. The crystallinity of CRS was 34.9 ± 0.3%, which was similar to CS (37.3 ± 0.6%), MS (34.3 ± 0.6%), WS (29.9 ± 1.0%), and SPS (37.1 ± 0.6%) (Figure 4B). However, this was not consistent with the significantly lower short-range ordered structure (Figure 2E,F). FTIR-derived short-range ordered structure is generally applied to compare how the crystallinity of native starch changes after modifications. Previous studies have proved that the crystallinity degree of native starches obtained by the FTIR and XRD methods is not correlated [48]. The crystallinity level of CRS found in the current study was in the middle compared with previous reports from strain 137C [27,28] and is significantly higher than other microalgal starches [39,45]. This indicates that CRS may exhibit greater resistance to enzymatic digestion.

### 2.5. Granular Structure

**Figure 5:**
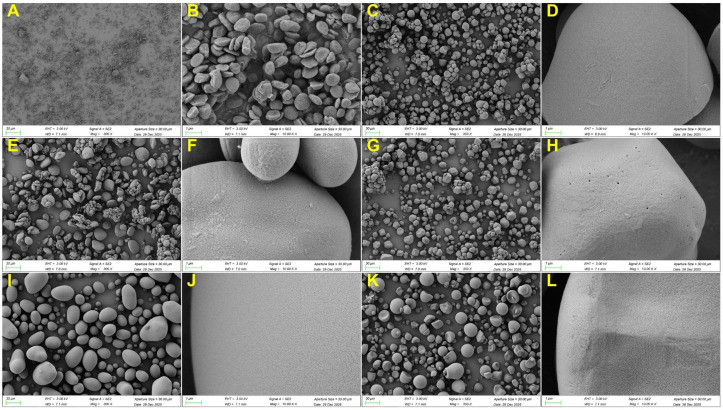
SEM image of *Chlamydomonas reinhardtii* starch (**A**,**B**), cassava starch (**C**,**D**), wheat starch (**E**,**F**), maize starch (**G**,**H**), potato starch (**I**,**J**), and sweet potato starch (**K**,**L**). (**A**,**C**,**E**,**G**,**I**,**K**) were taken at amplification 300×, and (**B**,**D**,**F**,**H**,**J**,**L**) were taken at amplification 10,000×.

**Figure 6:**
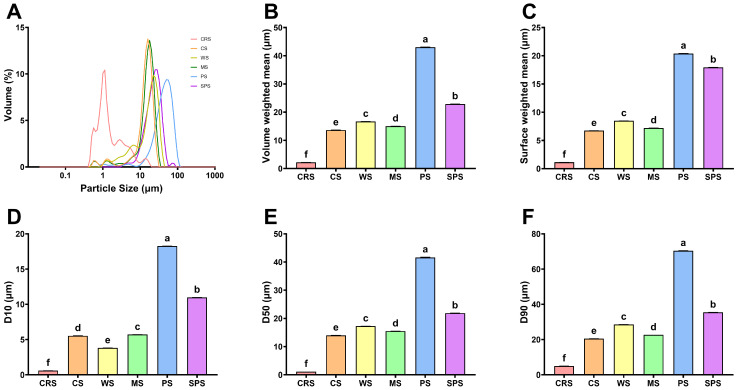
Particle size distribution (**A**), volume weighted mean diameter (**B**), surface weight mean diameter (**C**), D10 (**D**), D50 (**E**), and D90 (**F**) of *Chlamydomonas reinhardtii* starch (CRS), cassava starch (CS), wheat starch (WS), maize starch (MS), potato starch (PS), and sweet potato starch (SPS). Bars that do not share the same lowercase letter are significantly different (*p* < 0.05).

The periodic arrangement of lamellae gives rise to the semi-crystalline growth rings, which further consolidate into the complex architecture of the starch granule [49]. The granular structure differences were analyzed by scanning electron microscope (SEM) (Figure 5) and particle size analyzer (Figure 6). Figure 5A,B showed that CRS granule morphology was irregular, like a red blood cell, consistent with previous studies [28,29]. Starch from other microalgae, such as *Chlorella sorokiniana,* had a similar morphology to CRS [39,45,50]. The morphology of CRS differed from that of other starch samples. CS, PS, and SPS granules were spherical, hemispherical, or ellipsoidal. MS granules were polyhedral, and WS granules exhibited a lenticular shape. The size of the CRS granule was small but with some aggregates between 10 and 30 μm (Figure 5A). Particle size distribution revealed that CRS had several peaks with the strongest peak at about 1 μm (Figure 6A). The D[4,3] (volume weighted mean diameter) and D[3,2] (surface weighted mean diameter) were 2.2 and 1.1 μm (Figure 6B,C), respectively, similar to other microalgal starch [29,39]. D10, D50, and D90 represented the particle sizes that 10%, 50%, and 90% of the starch granules are smaller than, respectively. All these data of CRS were significantly smaller than those of other starch samples (Figure 6D–F), consistent with the gradually changed distribution of CRS in Figure 6A. The microalgal starch size was way smaller than terrestrial plant starch, which is due to the limitation of microalgal cell size. Potato possessed the largest D[4,3] and D[3,2], and the largest granule size was over 100 μm. The aggregation of CRS may be the result of high-pressure homogenization, lyophilization, and grinding processes [51,52]. While there was no large aggregate (>20 μm) detected in particle size distribution analysis (Figure 6A), indicating the breakdown of aggregate after dispersion in water. Thus, the aggregation of CRS was more likely temporally formed during lyophilization and grinding. Some granule integrity was also observed in CRS (Figure 5B). Compared with previous studies, CRS morphology in the current research exhibited a rougher surface and more fractures [29], indicating improper pressure or time of high-pressure homogenization.

### 2.6. Gelatinization Property

**Figure 7:**
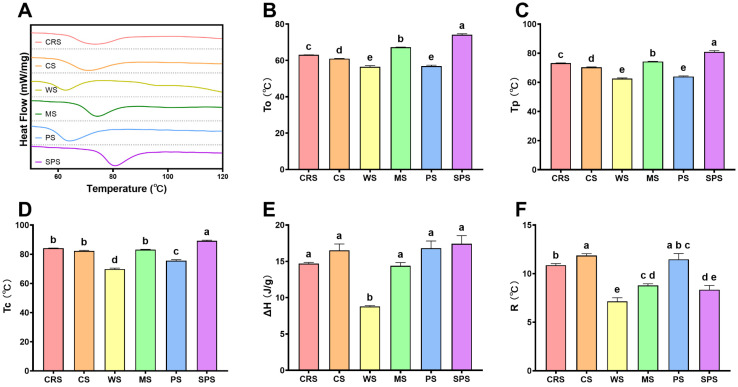
Heat flow curve (**A**), onset (**B**), peak (**C**), and conclusion (**D**) temperature, enthalpy (**E**), and gelatinization temperature range (Tc-To) (**F**) of *Chlamydomonas reinhardtii* starch (CRS), cassava starch (CS), wheat starch (WS), maize starch (MS), potato starch (PS), and sweet potato starch (SPS). Bars that do not share the same lowercase letter are significantly different (*p* < 0.05).

Starch gelatinization and pasting properties are primary physicochemical properties to determine its industrial application [53]. A differential scanning calorimeter (DSC) was applied to analyze CRS gelatinization with excess water. The heat flow curve of CRS was presented in Figure 7A. It exhibited one broad (Figure 7F) but shallow peak ranging from 63.0 to 84.1 °C with a peak center at 73.2 °C. The onset temperature (To) of CRS in the current study was similar to that of the previous study, while the peak temperature (Tp) and conclusion temperature (Tc) were higher [28]. The Tp and Tc of CRS in this study were also higher than those of other microalgal starches [39,46]. The broad DSC endotherm indicated a higher heterogeneity in the CRS granule. The heterogeneity may come from the smaller starch granule [33] or structural alteration due to high-pressure homogenization extraction (Figure 5 and Figure 6). The gelatinization temperature of CRS was intermediate, situated between the values observed for terrestrial starches such as CS, WS, MS, PS, and SPS (Figure 7B–D). However, the enthalpy of CRS was relatively low (not significant) compared with other starches (Figure 7E), which was consistent with the lower short-range ordered structure (Figure 2E,F). Gelatinization refers to the process by which raw starch transforms into a cooked, digestible form through the combination of water and heat [54]. The lower enthalpy of CRS indicated it was more energy efficient in industrial applications.

### 2.7. Pasting Property

**Figure 8:**
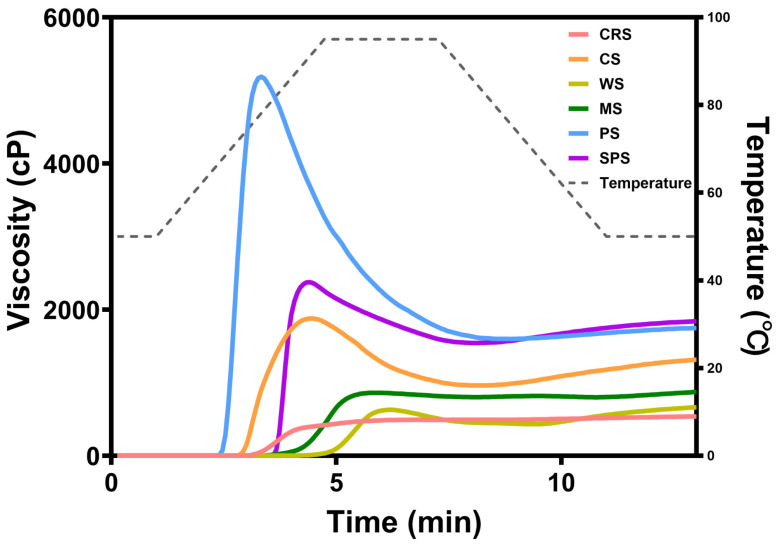
Pasting curve of *Chlamydomonas reinhardtii* starch (CRS), cassava starch (CS), wheat starch (WS), maize starch (MS), potato starch (PS), and sweet potato starch (SPS).

Pasting properties of starch samples were determined by rapid viscosity analyzer (RVA). The viscosity curves of starch samples during temperature changes are shown in Figure 8. The peak viscosity and final viscosity of CRS were relatively low compared with other starches. Table 1 presents the pasting parameters of starch samples, and the peak viscosity, final viscosity, trough viscosity, breakdown, and setback of CRS were statistically lowest of all starches. No clear decrease in viscosity was found in the curve of CRS because the breakdown was only 8 cP, indicating high thermostability and shear resistance during hydrothermal treatment [39]. This pattern was consistent with previous studies focusing on *Chlorella* starch [39,45]. Previous studies also found that cereal starch with small granules exhibited a similar pattern to microalgal starch [39,45]. PS, with the largest granule, exhibited the highest peak viscosity, followed by SPS, which possessed the second largest granules among the samples. This indicated that the peak viscosity of CRS was affected by granular size. The peak viscosity as measured by RVA is a tradeoff between the swelling of granules and the disintegration of swelled granules due to rotating paddles [55]. Extant literature indicates that starch granules of a larger size exhibit significantly higher peak and final viscosities compared to their smaller counterparts derived from the same botanical source [56].

### 2.8. Digestibility

**Figure 9:**
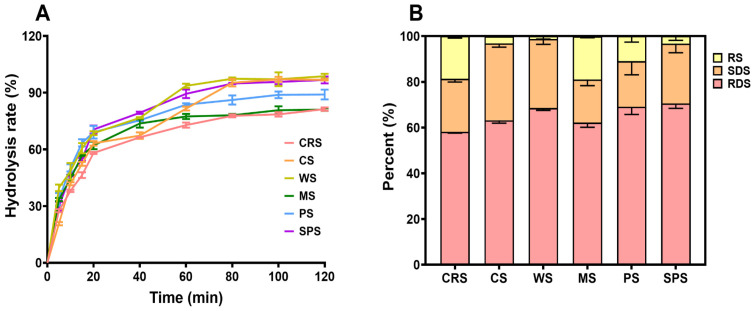
Hydrolysis curve (**A**) and rapidly digestible starch (RDS), slowly digestible starch (SDS), and resistant starch (RS) content (**B**) of *Chlamydomonas reinhardtii* starch (CRS), cassava starch (CS), wheat starch (WS), maize starch (MS), potato starch (PS), and sweet potato starch (SPS).

The digestibility of CRS was determined by the Englyst method [57]. The starch digestibility has drawn great attention in the starch research area due to its influence on type 2 diabetes [58]. A high RS content and low RDS content starch is preferred for individuals with metabolic disorders. Figure 9A presents the hydrolysis curve of starch samples after the addition of the enzyme mixture. The CRS showed the lowest hydrolysis rate at almost all time points and was close to MS at the end of the test. The RDS content of CRS was relatively lower than that of other starch samples, and the RS content was significantly higher than CS, WS, and SPS (Figure 9B). Previous studies of microalgal starch characterized it as easily digestible. Jiang et al. [39] determined the RDS of raw and cooked *Chlorella sorokiniana* starch and found that it contained a higher amount of RDS and less RS than PS, WS, and MS. Shen et al. [50] reported that the RS content of *Tetraselmis subcordiformis* starch was lower than that of normal corn starch. Similar lower RS content results of *Chlorella* sp. starch were reported by Chi et al. [45]. The current study found that cooked CRS was more resistant to enzyme digestion, which was different from other cooked microalgal starches. The starch digestibility is determined by enzyme accessibility to starch molecular chains. In uncooked starch, the enzyme cannot access the internal chains due to the granule structure, crystalline structure, and so on [59]. After gelatinization, the granule and semi-crystal structure melted, and the starch digestibility usually increased after cooking [39]. Thus, the original structures cannot reflect the exact condition of starch molecules in solution [24]. When the starch solution cools down, retrogradation starts, and inter- and intra-molecular single helices and double helices are formed. Furthermore, long-time storage allowed the recrystallization of starch molecules [60]. Considering the short time between gelatinization and digestion in this experiment, the amylose structure reorganization was the major retrogradation that happened [61]. However, the low amylose content and resulting low retrogradation of CRS could not explain the high resistant starch content (Figure 1C). The high thermal stability of CRS, as evidenced by the RVA profile (Figure 9), suggests a strong likelihood that the starch granules retain their semi-crystalline integrity even after cooking [62], making CRS more resistant to digestion. In addition, increased content of α-1,6 linkages usually led to a reduction in RDS content [63]. The CRS had a significantly higher degree of branching compared with terrestrial starch (Figure 1B), indicating a lower RDS content and higher RS content.

## 3. Conclusions

This study provided a comprehensive characterization of the multiscale structure and functional properties of starch from model microalgae *Chlamydomonas reinhardtii*. Direct comparison of CRS with major terrestrial plant starches was established. At the structural level, CRS exhibited an exceptionally high degree of branching (18.6%), and MS presented the lowest degree of branching. CRS showed thinner crystalline lamellae (9.29 nm) compared with other A-type crystalline starches. A significantly higher crystallinity of CRS was detected compared to PS. CRS exhibited a unique red blood cell-like morphology with an average diameter of about 1 μm, while other starches presented spherical, oval, or polygonal morphology with a diameter ranging from 10 to 50 μm. These granular features, constrained by the microalgal chloroplast’s spatial limits, significantly differentiate CRS from the larger ellipsoidal or polyhedral granules of crops like potato or maize.

These structural characteristics translated into distinct physicochemical and nutritional advantages. CRS possessed an intermediate gelatinization temperature but a lower enthalpy, suggesting a stable yet energy-efficient crystalline structure for industrial processing. Its pasting profile is uniquely marked by low viscosity and nearly no decrease, which indicates its stability under high-temperature or high-shear conditions. Most notably, from a nutritional perspective, cooked CRS demonstrates a significantly lower hydrolysis rate and higher RS content than common terrestrial starches. This reduced digestibility, driven by its complex branching and superior thermostability, positions CRS as a promising low-glycemic index (GI) functional ingredient. Ultimately, these findings provided a robust scientific basis for utilizing *C. reinhardtii* as a sustainable “green yeast” bio-resource for the next generation of functional foods.

## 4. Materials and Methods

### 4.1. Materials

Microalgae strain *C. reinhardtii* FACHB-2218 was obtained from the Freshwater Algae Culture Collection at the Institute of Hydrobiology, Chinese Academy of Science. Cassava starch (CS), wheat starch (WS), maize starch (MS), potato starch (PS), and sweet potato starch (SPS) were purchased from Xinxiang Liangrun Whole Grain Food Co., Ltd. (Henan, China). Amyloglucosidase and α-amylase were obtained from Shanghai Yuanye Bio-Technology Co., Ltd. (Shanghai, China) and Sigma-Aldrich (St. Louis, MO, USA), respectively. The glucose kit (cat number TC0711) was obtained from Beijing Leagene Biotechnology Co., Ltd. (Beijing, China). The amylose (from potato, cat# S11003) and amylopectin (from maize, cat# S11002) standards were purchased from Shanghai Yuanye Bio-Technology Co., Ltd. (Shanghai, China). All other chemicals used and reagents were of analytical grade.

### 4.2. Cultivation of C. reinhardtii FACHB-2218

**Figure 10:**
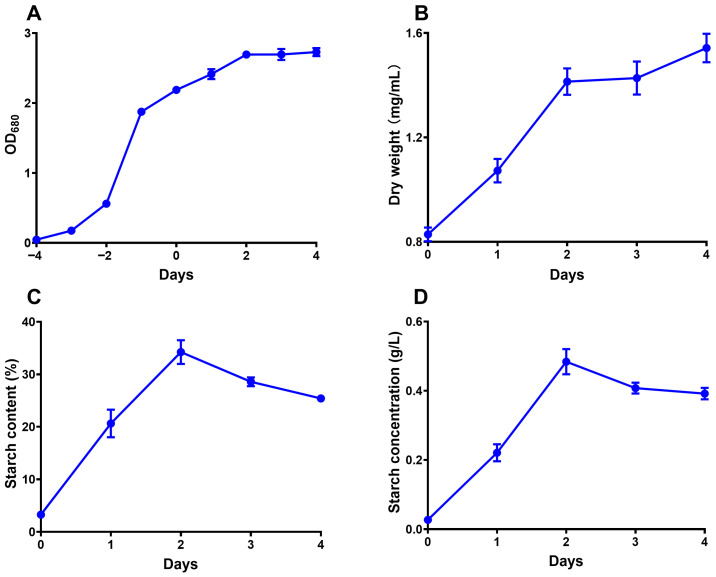
The OD (**A**), biomass (**B**), starch content of biomass (**C**), and starch concentration of medium (**D**) change during the cultivation period.

The seed of *C. reinhardtii* FACHB-2218 was inoculated in an Erlenmeyer flask containing 20 mL of tris-acetate-phosphate (TAP) medium [64] and cultivated for 4 days. Then, the culture was inoculated into 8 L of fresh TAP (with nitrogen) and cultivated for another 4 days. The medium was then switched from nitrogen repletion to nitrogen depletion to induce starch accumulation, and the day of switching was designated as day 0. The microalgal cells were harvested on day 4 by centrifugation and stored at −20 °C. All cultivations were performed under 25 °C with illumination of 150 μmol·m^−2^·s^−1^ in Zhichu ZQLY-180G light oscillation incubator (Shanghai, China). Air was used for agitation and carbon dioxide supply. The optical density (OD) at 680 nm of the cultivation medium was recorded throughout the cultivation period by an Metash X-6 optical spectrometer (Shanghai, China). The biomass (dry weight) of 20 mL of cultivation medium was measured by a Precisa XM 60-HR rapid moisture analyzer (Dietikon, Switzerland) starting from day 0. The OD, biomass, and starch content changes during the cultivation period are shown in Figure 10.

### 4.3. Starch Analysis

The total starch content of *C. reinhardtii* was analyzed according to Fan et al. [14] with minor modification. Briefly, 10 mL culture was centrifuged at 4000× *g* for 5 min, and the pellet was washed with 1 mL methanol for five cycles. Then the pellet was resuspended with 0.8 mL 0.2 mol/L KOH and incubated at 95 °C for 1 h for starch solubilization. Further, the mixture was centrifuged at 4000× *g* for 5 min to remove the precipitate. An amount of 0.2 mL 6 mol/L HCl was added to the supernatant and incubated at 95 °C for 2 h for starch degradation. After neutralization with 2 mol/L Tris base and centrifugation for 5 min at 12,000× *g*, the supernatants were used for glucose quantification using a glucose kit. Starch content was calculated as 0.9-fold of glucose content.

### 4.4. Starch Extraction

According to the method developed by Six et al. [46], CRS was extracted by the Antuos, AH-MINI PLUS high-pressure homogenizer (Suzhou, China) with some modifications. The microalgal cells harvested were diluted to 10% dry weight (*w*/*v*) in water, and 200 mL of the diluted mixture was added to the homogenizer for each run. After continuous homogenization under 1200 bar and 4 °C for 2 min (about 6 cycles), the cell lysate was centrifuged for 10 min at 4000× *g*. The bottom white precipitate was recovered and washed with deionized water for three cycles. Then the purified starch was lyophilized and stored at room temperature.

### 4.5. SEM Analysis

The SEM analysis was performed using a ZEISS GeminiSEM 360 Scanning Electron Microscope (Oberkochen, Germany). Samples were fixed using double-faced tape and sputter-coated with gold before observation. Images were taken at an accelerating voltage of 3 kV with 300× and 10,000× magnification.

### 4.6. Particle Size Distribution

The particle sizes of starch samples were analyzed by Malvern Mastersizer 2000 (Worcestershire, UK). Deionized water was chosen as the dispersion solvent. The refractive index for water and starch was 1.33 and 1.53, respectively.

### 4.7. XRD Analysis

The X-Ray diffraction patterns of starch samples were analyzed using a Bruker D8 ADVANCE X-ray diffractometer (Billerica, MA, USA) at a voltage of 40 kV. The diffraction was detected in an angular range of 5–40° (2θ) with a scanning speed of 6° (2θ)/min and a step size of 0.02° (2θ). The background correction was carried out before sample spectrum collection. The relative crystallinity of each sample was calculated with MDI Jade software (version 6.5).

### 4.8. SAXS Analysis

The lamellar structure of starch samples was measured through a Xenocs Xeuss 3.0 small-angle X-ray scattering instrument (Grenoble, France) under a wavelength, voltage, and current of 0.135 nm, 70 kV, and 3.5 mA, respectively. Starch samples were dispersed in deionized water with a concentration of 1% (*w*/*v*) and kept at 4 °C overnight. The samples were centrifuged at 4000× *g* to remove the excess water before the test. I-q curve was plotted between 0.2 and 1.5 nm^−1^, and lamellar thickness was calculated following Equation (1).

(1) $$D=\frac{2\pi }{q_{0}}$$

where D is the average thickness of the lamellar structure, and q_0_ is the peak q value in the I-q curve.

### 4.9. FT-IR Analysis

The FTIR analysis was conducted using a Thermo Scientific Nicolet iS20 FTIR Spectrometer (Waltham, MA, USA). Scanning in absorbance mode was performed with 32 scans at a resolution of 4 cm^−1^ in the range of 4000–400 cm^−1^. The absorbance was deconvoluted, and the ratio of peak absorbance at about 1047, 1022, and 995 cm^−1^ was calculated as R_1047/1022_ and R_995/1022_.

### 4.10. ^1^H-NMR Analysis

The ^1^H NMR analysis was performed using an Bruker Avance III 500M Spectrometer (Billerica, MA, USA) at 500 MHz. Briefly, 2 mg of starch samples were dissolved in 0.6 mL of DMSO-d_6_ at 70 °C before spectrum recording. The chemical shift was calibrated with the solvent peak at 2.50 ppm, and peak areas were calculated with MestReNova software (version 14). Peaks at 5.10 and 4.91 ppm were recognized as α-1,4 and 1,6 glycosidic bonds, and the degree of branching was calculated as the ratio of peak area at 4.91 ppm to the sum of peak area at 5.10 and 4.91 ppm.

### 4.11. Amylose Content

The amylose content was determined by the iodometric method following ISO 6647-1:2020 [65]. The calibration curve was constructed of amylose ranging from 0 to 50%.

### 4.12. DSC Analysis

The gelatinization properties of starch samples were evaluated using a TA Discovery 2500 (New Castle, DE, USA) instrument. Starch sample (10 mg) was mixed with 20 μL ultrapure water in an aluminum pan. Samples were sealed and equilibrated at 4 °C overnight. The sample was heated from 30 to 120 °C with a heating rate of 10 °C/min. Gelatinization temperature and enthalpy change were calculated with TRIOS software (Version 4.0).

### 4.13. Rapid Viscosity Analysis

The pasting properties of starch samples were analyzed by the Perten RVA-TecMaster rapid viscosity analyzer (Springfield, IL, USA). The tests were performed according to Xu et al. [66]. Briefly, two grams of starch samples were mixed with 25 mL of deionized water. The mixture was equilibrated at 50 °C for 1 min, then ramped up to 95 °C at 12 °C/min, holding at 95 °C for 2.5 min, cooling to 50 °C at 12 °C/min, and holding at 50 °C for 2 min at last. Stirring speed was set to 960 rpm for the initial 10 s, then adjusted to 160 rpm for the remainder of the run.

### 4.14. Evaluation of Starch Digestibility

The hydrolysis curve, rapidly digestible starch (RDS), slowly digestible starch (SDS), and resistant starch (RS) were determined according to a previous report [52]. Briefly, 50 mg samples were dispersed in 2.5 mL sodium acetate buffer (pH 5.2) and maintained at a 95 °C water bath for 30 min with magnetic stirring. The cooked suspension was cooled down to 37 °C, and 500 μL enzyme solution (80 mg of α-amylase and 60 mg of amyloglucosidase dissolved in 10 mL of sodium acetate buffer) was added to the suspension. After the addition of the enzyme solution, the mixture was then kept at 37 °C, and 20 μL of suspension was transferred into 1 mL of ethanol at 0, 5, 10, 15, 20, 40, 60, 80, 100, and 120 min. The mixture of suspension and ethanol was vortexed and centrifuged at 4000× *g* for 5 min. The glucose concentration of the supernatant was measured using a glucose kit. The content of RDS, SDS, and RS was calculated according to the following equation.

(2) $$RDS=\left(G_{20}-FG\right)\times 0.9$$

(3) $$SDS=(G_{120}-G_{20})\times 0.9$$

(4) RS=TS−RDS−SDS

where G_20_ and G_120_ are the glucose mass determined at 20 and 120 min of digestion, respectively; FG is the glucose mass determined at 0 min of digestion; and TS is the total starch dry weight mass.

### 4.15. Statistical Analysis

Data were expressed as mean ± standard deviation and analyzed using Graphpad Prism software (version 10.2.3). Significant difference (*p* < 0.05) between groups was evaluated by one-way ANOVA. Dunnett’s T3 test was used for multiple comparisons. All results were confirmed from at least three independent experiments. All results of CRS were confirmed from at least three independent extractions from separate cultures.

## Figures and Tables

**Table 1:** Pasting parameters of starch samples.

	Peak Viscosity (cP)	Trough Viscosity (cP)	Final Viscosity (cP)	Breakdown (cP)	Setback (cP)
CRS	493 ± 4 ^f^	484 ± 3 ^d^	542 ± 4 ^e^	8 ± 1 ^e^	58 ± 5 ^d^
CS	1881 ± 17 ^c^	966 ± 7 ^b^	1329 ± 13 ^b^	915 ± 10 ^b^	363 ± 15 ^a^
WS	637 ± 16 ^e^	437 ± 13 ^d^	671 ± 17 ^d^	200 ± 3 ^d^	233 ± 4 ^bc^
MS	863 ± 13 ^d^	802 ± 5 ^c^	874 ± 16 ^c^	61 ± 9 ^e^	72 ± 11 ^d^
PS	5278 ± 85 ^a^	1574 ± 21 ^a^	1745 ± 29 ^a^	3704 ± 92 ^a^	171 ± 23 ^cd^
SPS	2375 ± 17 ^b^	1541 ± 10 ^a^	1852 ± 9 ^a^	834 ± 7 ^c^	311 ± 14 ^ab^

Values are presented as the mean ± SD. Values that do not share the same lowercase letter are significantly different (*p* < 0.05).

## Data Availability

The original contributions presented in this study are included in the article. Further inquiries can be directed to the corresponding authors.
